# Unveiling an NMR-Invisible Fraction of Polymers in Solution by Saturation Transfer Difference

**DOI:** 10.1021/acsmacrolett.1c00628

**Published:** 2021-11-10

**Authors:** Ramon Novoa-Carballal, Manuel Martin-Pastor, Eduardo Fernandez-Megia

**Affiliations:** † Centro Singular de Investigación en Química Biolóxica e Materiais Moleculares (CIQUS), Departamento de Química Orgánica, Universidade de Santiago de Compostela, Jenaro de la Fuente s/n, 15782 Santiago de Compostela, Spain; ‡ Unidade de Resonancia Magnética, Área de Infraestructuras de Investigación, CACTUS, Universidade of Santiago de Compostela, 15782 Santiago de Compostela, Spain

## Abstract

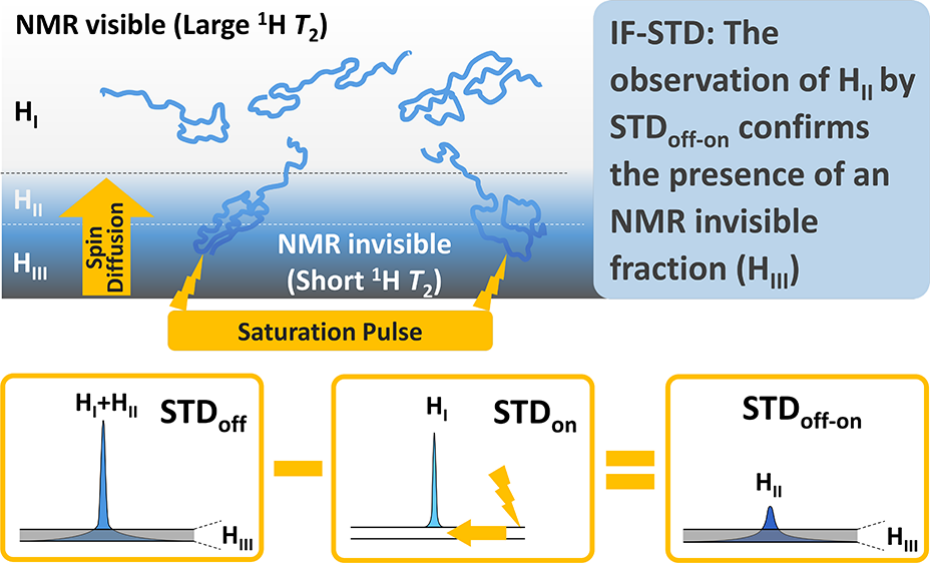

The observation of signals in solution NMR requires nuclei with sufficiently large transverse relaxation times (*T*_2_). Otherwise, broad signals embedded in the baseline afford an invisible fraction of nuclei (IF). Based on the STD (saturation transfer difference) sequence, IF-STD is presented as a quick tool to unveil IF in the ^1^H NMR spectra of polymers. The saturation of a polymer in a region of the NMR spectrum with IF (very short ^1^H *T*_2_) results in an efficient propagation of the magnetization by spin diffusion through the network of protons to a visible–invisible interphase with larger ^1^H *T*_2_ (STD_on_). Subtracting this spectrum from one recorded without saturation (STD_off_) produces a difference spectrum (STD_off-on_), with the nuclei at the visible–invisible interphase, that confirms the presence of an IF. Analysis of a wide collection of polymers by IF-STD reveals IF more common than previously thought, with relevant IF figures when STD > 0.4% at 750 MHz. A fundamental property of the IF-STD experiment is that the signal is generated within a single state comprising polymer domains with different dynamics, as opposed to several states in exchange with different degrees of aggregation. Contrary to a reductionist visible–invisible dichotomy, our results confirm a continuous distribution of nuclei with diverse dynamics. Since nuclei observed (edited) by IF-STD at the visible–invisible interphase are in close spatial proximity to the IF (tunable with the saturation time), they emerge as a privileged platform from which gaining an insight into the IF itself.

In nuclear magnetic resonance (NMR), series of scans are implemented to improve the signal-to-noise ratio. Each scan is followed by a delay, where the spin systems return to equilibrium by longitudinal (*z*-axis) and transverse (*xy*-plane) relaxation. The transverse component of the magnetization decays to zero according to eq 1,
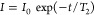
1where *I*_0_ is the intensity of the magnetization at time *t* = 0, and *T*_2_ is the transverse relaxation time of each nucleus.^1^

The observation of signals in solution NMR requires nuclei with sufficiently large *T*_2_. Otherwise, the inverse proportionality between *T*_2_ and the spectral line width results in signals so broad they remain embedded in the baseline (NMR-invisible or silent).^2^ This is the case for macromolecular systems like vesicles,^3^ polyelectrolyte complexes,^4^ and gels.^5^ Conversely, NMR-invisible fractions (IF) are seldom observed in polymers, with few reports restricted to charged polysaccharides.^6−9^ The widespread belief that IF are uncommon in solution NMR, along with the counterintuitive nature of detecting anything invisible, have sunk the IF of polymers into oblivion.

Our laboratory has unveiled an IF in the ^1^H NMR of chitosan (CS, Figure 1A)^10^ with IF% (percentage of signal not detected) as high as 50% in the semidilute regime, depending on structural [molecular weight (MW), degree of acetylation (DA)] and experimental (concentration, temperature, pH, magnetic field) parameters.^10^ Although overlapped/aggregated CS chains display short ^1^H *T*_2_ values (10–25 ms at 500 MHz),^11,12^ the existence of an IF at concentrations as low as 1 mg/mL (close to the overlap concentration) was completely unexpected.^13^

**Figure 1:**
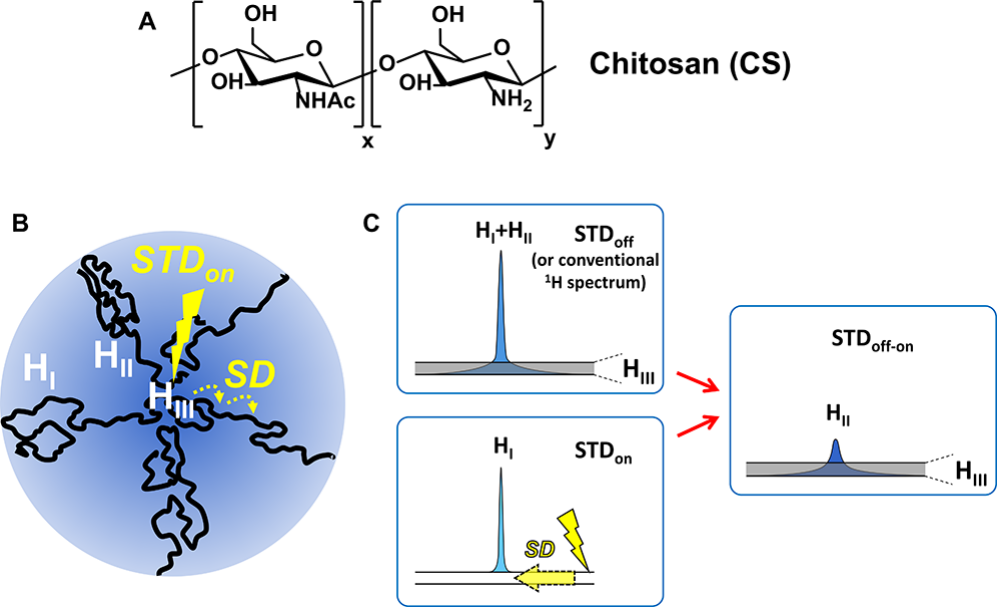
(A) Structure of chitosan. (B) Schematic representation of regions with different mobility in a polymer in solution: the line width of proton signals in the most flexible H_I_ region (large ^1^H *T*_2_) is much narrower than in the more rigid H_II_ (visible–invisible interphase) and H_III_ (invisible fraction, IF; very short ^1^H *T*_2_). (C) IF-STD experiment: although the intensity of protons in region H_III_ (IF) is below the limit of detection, their saturation in a STD_on_ spectrum is transferred through spin diffusion to protons in the H_II_ region (visible–invisible interphase observed in the STD_off-on_), but does not reach region H_I_. Note that nuclei observed in the STD_off_ and conventional NMR experiments correspond to H_I_ + H_II_.

Since NMR data acquired for polymers in solution in the presence of an IF unlikely describe the overall sample, the necessity of a straightforward tool for the identification of IF was evident. Saturation transfer difference (STD)^14,15^ lays among the most potent NMR experiments to study the interaction between small ligands and proteins^16^/receptors.^17,18^ It relies on an extremely efficient propagation of the magnetization across the entire network of protons in large complexes by spin diffusion.^19^ The selective irradiation of a receptor with a saturation radiofrequency on a region of the spectrum free of ligand protons, but with broad signals of the receptor embedded in the baseline, results in saturation efficiently transferred through the receptor to the nuclei of the complexed ligands. Since ligands with a relatively weak-affinity for the receptor will be released in the NMR time scale to allow recording of the spectrum while still saturated (on-resonance experiment; STD_on_), subtracting this spectrum from one recorded without saturation (off-resonance, STD_off_) produces a difference spectrum (STD_off-on_) in which only the signals of the binding ligands remain. The intensity of nonbinding ligands is identical in the on- and off-spectra so, they cancel out in the STD_off-on_. The STD factor is expressed by eq 2.
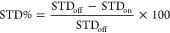
2Herein, we propose STD for unveiling the IF of polymers (IF-STD, Figure 1B,C). The selective saturation of a polymer in a region of the NMR spectrum free of signals should afford a nil STD_off-on_ unless broad signals embedded in the baseline (IF with very short ^1^H *T*_2_, region H_III_ in Figure 1B,C) transfer saturation to a visible–invisible interphase (region H_II_) with larger ^1^H *T*_2_ and faster dynamics. Spin diffusion is a time-dependent phenomenon with a length scale (*L*) that correlates to the longitudinal relaxation time (*T*_1_) as follows (eq 3):^20^
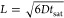
3*D* is the spin diffusion coefficient (typically 0.1 nm^2^/ms)^21^ and *t*_sat_ is the saturation time of the IF-STD experiment (constrained to a maximum length for *t*_sat_ = *T*_1_). As for IF-STD experiments carried out with a *t*_sat_ of 1–3 s (assuming *t*_sat_ ≤ *T*_1_), the spin diffusion is expected to propagate within the polymer up to a length of ∼25–42 nm; the appearance of H_II_ signals in the STD_off-on_ spectrum would not only confirm the presence of an IF, but also turn the visible–invisible interphase into a privileged viewing platform to gain an insight into the IF itself.

IF-STD resembles the dark state exchange saturation transfer (DEST) described by Clore to visualize proteins and peptides in exchange with much larger macromolecular assemblies.^21−23^ A major difference between IF-STD and DEST is that the latter relies on a chemical exchange mechanism between at least two states in solution with different degree of aggregation (i.e.; molecular tumbling), interconverting on an intermediate-to-slow time scale. Conversely, the transfer of magnetization in IF-STD starts from the saturated nuclei at the IF of a polymer and reaches the visible signals by spin diffusion,^19^ with the whole process occurring within a single state populated by nuclei with different dynamics (Figure 1B). IF-STD also differs from the chemical exchange saturation transfer (CEST),^24^ a DEST-related experiment focused on the chemical exchange on a slow time scale between visible and dark state species with significantly different chemical shifts. Another subtle distinction is that application of IF-STD does not require the prior knowledge of an IF.

IF-STD was first assessed with CS (MW 80 kDa, DA 14%). An 8% IF was determined for this sample (10 g/L, 750 MHz) by analyzing the relative integral between the acetyl signal and that of an external reference in a series of spectra recorded at increasing temperatures (Figure S3 and SI). The effect of saturating at different regions of the spectrum is shown in Figure 2. As expected, when the on-saturation was placed at 12 ppm, far away from any polymer resonance, the resulting STD_off-on_ spectrum showed no signals, confirming the absence of an IF in this region of the spectrum (Figure 2A). Conversely, if the on-saturation was applied closer to the polymer resonances (7.5, 0.54, −0.5 ppm), STD_off-on_ signals revealed an IF in these regions. Higher STD factors were observed the closer the on-saturation to the polymer signals. When various *t*_sat_ were tested, higher STD factors were obtained for the longer times (Figures 2B, S4, and S5). For experiments recorded at different magnetic fields and temperatures, higher STD factors were seen for the lower fields and lower temperatures (Figures S4 and S5), consistent with the proposed spin diffusion mechanism. Having confirmed the existence of an IF by STD, we proceeded to evaluate the effect of the MW and concentration on the intensity of IF-STD. Indeed, both parameters were identified in our previous report as key elements governing the appearance of an IF for CS.^10^ As expected (Figure 2C,D; spectra in Figures S6 and S7), increasing the MW and concentration resulted in higher STD factors associated with the appearance of an IF due to overlapping/entangled CS chains with slower dynamics and lower ^1^H *T*_2_.^12,13^ Lastly, the influence of the saturation pulse bandwidth (BW, see the SI) on the intensity of IF-STD was analyzed between 110 and 300 Hz (Figure S8). It was confirmed that the shorter the BW, the more selective the on-saturation pulse and so, the smaller the STD factors. A BW of 130 Hz (equivalent to 0.17 ppm at 750 MHz) was selected as a balance between pulse selectivity and IF-STD sensitivity (only 96–128 scans needed).

**Figure 2:**
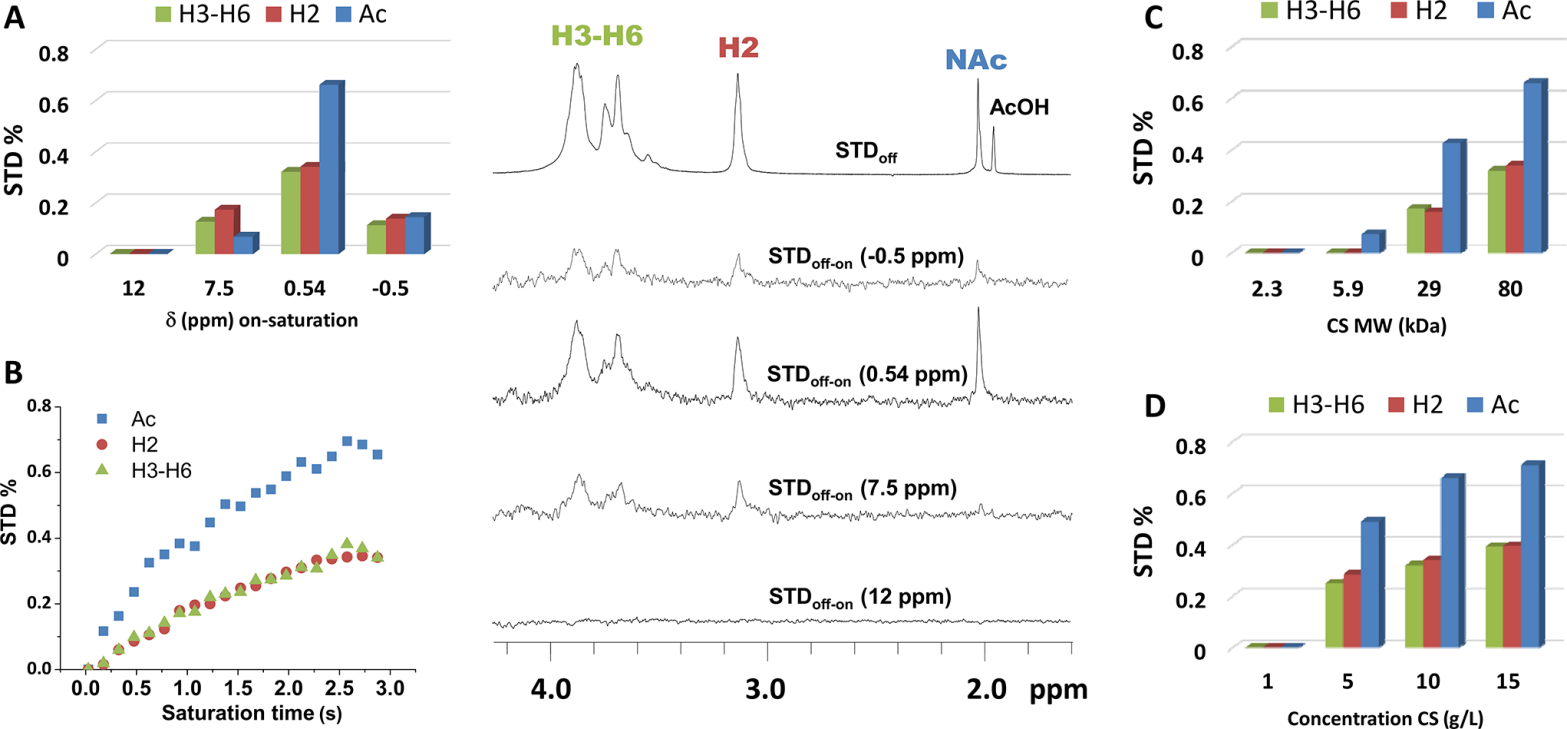
Central panel: IF-STD spectra (750 MHz, 298 K, *t*_sat_ 3 s, BW 130 Hz) of CS (80 kDa, DA 14, 10 g/L in pD 4.5 acetate buffer). STD_off-on_ (×100) with on-saturation at −0.5, 0.54, 7.5, and 12 ppm. Left panel: STD factor as a function of the on-saturation ppm (A) and *t*_sat_ (on-saturation at 0.54 ppm, B). Right panel: STD factor (on-saturation at 0.54 ppm) as a function of the MW (C) and concentration of CS (D). Note that “H3–H6” includes all H3–H6 plus H2 of *N*-acetyl glucosamine, while “H2” refers to H2 of glucosamine. Interestingly, IF-STD spectra lack the residual nondeuterated signal of the acetate buffer as it does not interact with the IF.

To explore the scope of IF-STD as technology to ascertain the presence of an IF in the ^1^H NMR spectra of polymers, a collection of natural and synthetic polymers [polysaccharides, polypeptides, acrylates and other vinyl polymers, polyethylenimine (PEI), polyethylene glycol (PEG), and a globular poly(propyleneimine) (PPI) dendrimer] was studied by IF-STD in D_2_O (10 g/L, 750 MHz; Figure 3 and Table 1). Samples were saturated for 3 s in a region of the spectra devoid of protons at −1.5 ppm from the lowest ppm visible signal, selected as region of interest [the 130 Hz BW affords a 1.41 ppm gap (1.5–0.17/2) between the on saturation upper limit and the starting point of the visible signal]. Table 1 includes STD factors (integration ranges in Table S1), ^1^H *T*_2_ values, and IF% (Figures 3D and S3 and Supporting Information) for the signals analyzed. IF-STD spectra are given in Figure 3 and the SI.

**Figure 3:**
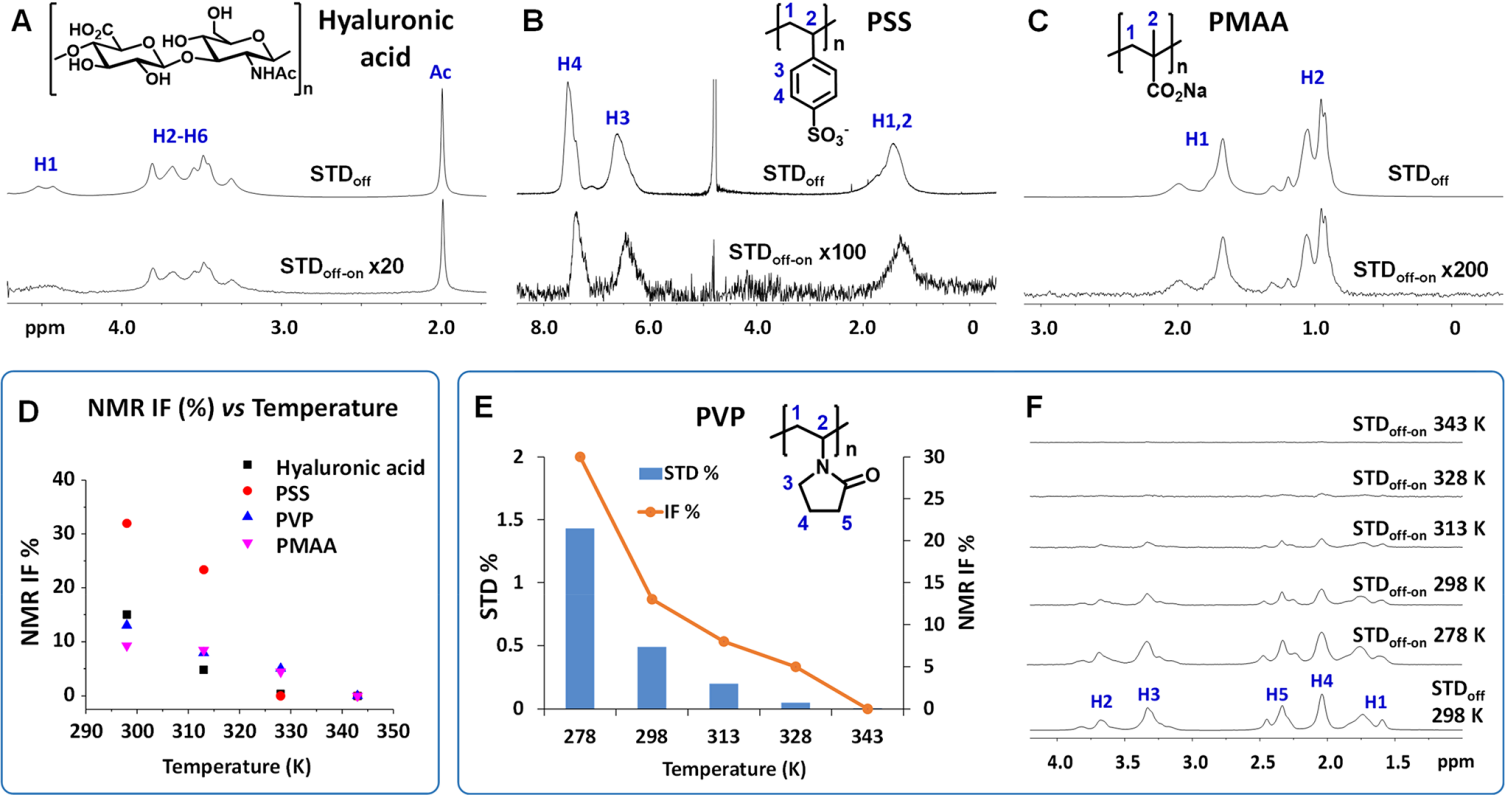
IF-STD spectra (750 MHz, 298 K, 10 g/L in D_2_O, on-saturation at −1.5 ppm from the lowest ppm visible signal) of hyaluronic acid 160 kDa (A), PSS 1000 kDa (B), PMAA 30 kDa (C), and PVP 360 kDa (F). Effect of the temperature on the IF of polymers (D). Effect of the temperature on the STD factor and IF of PVP. STD_off-on_ (×20) (E, F).

**Table 1:** STD Factors and IF of Polymers (750 MHz, 298 K, 10 g/L in D_2_O)^a^

polymer	MW (kDa)	^1^H *T*_2_ (ms)^b^	STD%^c^	NMR IF%^d^
hyaluronic acid	160	31	3.4	15
carrageenan	647	20	1.6	43
PSS	1000	8	1.1	32
chitosan (CS)	80	26	0.66	8
PVP	360	19	0.49(1.4)^e^	≥13
PMAA	30	21	0.43	≥9
ulvan	524	25	0.42	15
PAA (pD 7.0)	450	16	0.52	11
PAA (pD 3.0)	450	67	0.10(0.60)^e^	0
polyacrylamide	5000	80	0.12	nd
pullulan	788	100	0.10(0.16)^e^	0
pullulan	112	100	0	nd
pullulan	12	100	0	nd
PVA	306	98	0.06	nd
PGA	35	92	0.05(0.10)^e^	0
PGA	14	92	0	0
PLL	45	69	0.05(0.10)^e^	nd
PEG	10	400	0	0
PEI-branched	25	145	0	nd
PPI-G4	3.5	29–242	0.02–1.49^e^	nd

a STD factors, IF, and ^1^H *T*_2_ for the lowest ppm visible signal in the NMR spectrum of each polymer. CS dissolved in pD 4.5 buffer. PSS [poly(styrenesulfonate)], PVP [polyvinylpyrrolidone], PMAA [sodium poly(methacrylate)], PAA [poly(acrylic acid)], PVA [poly(vinyl alcohol)], PLL [poly-l-lysine], PGA [poly-l-glutamic acid], PEG [poly(ethylene glycol)], PEI [polyethyleneimine], PPI-G4 [poly(propylene imine) dendrimer of G4].

b Determined at 500 MHz; ^1^H *T*_2_ for CS and hyaluronic acid are mean values of all resonances.

c BW 130 Hz, *t*_sat_ 3 s, on-saturation at −1.5 ppm from the signal analyzed.

d Determined by integration relative to an external reference in a series of NMR spectra recorded at increasing temperatures (298–343 K).

e STD factor at 278 K.

We started analyzing by IF-STD the negatively charged, rigid polysaccharides, hyaluronic acid and carrageenan, both with a sizable IF previously described.^7,9^ In our hands, IF in the 15–43% range were revealed, accompanied by strong STD factors (STD > 1%, Table 1). Besides CS (8% IF, 0.66% STD), relatively high STD (>0.4%) were also obtained for polyelectrolytes with previously unknown IF, like PSS, ulvan, and the sodium salts of PMAA and PAA. Gratifyingly, an IF determination of these samples afforded figures higher than 9%, confirming the potential of IF-STD for the quick identification of IF in solution. Indeed, the stiffness of polyelectrolytes favors the appearance of an IF and strong IF-STD as demonstrated when comparing the 0.52% STD and 11% IF of PAA at pD 7 (anionic polyelectrolyte) with a negligible STD and no IF at pD 3.0 (protonated carboxylic acids, no electrostatic repulsion). Still, the presence of an IF is not restricted to polyelectrolytes. While the highly flexible PEG and PEI resulted in neither IF-STD nor IF, the more rigid PVP afforded a 0.49% STD and ≥13% IF. The high sensitivity of IF-STD is also demonstrated by examples without IF but weak STD factors (<0.15%), such as PGA and pullulan. Structurally related polymers like PVA, polyacrylamide, and PLL also displayed STD < 0.15%.

As seen for CS, IF-STD factors in Table 1 responded to the temperature and MW as the IF%. Either reducing the temperature (PVP, PAA, pullulan, PLL, PGA) or increasing the MW (pullulan, PGA) resulted in larger STD factors and growing IF (Table 1 and SI). Figure 3E,F shows a series of STD_off-on_ spectra for PVP at different temperatures that illustrate this trend. While null STD_off-on_ and IF were observed at 343 K, these steadily increased up to 1.4% STD and 30% IF on reducing the temperature down to 278 K (same effect for CS in Figure S5). Certainly, any variation of structural or recording parameters (MW, concentration, temperature, pH, magnetic field) leading to reduced polymer dynamics (lower ^1^H *T*_2_) will favor the appearance of an IF and intense IF-STD. Ultimately, despite the lack of a direct proportionality, STD% reveals as a good indicator of the presence of an IF (Table 1) with STD > 0.4% (associated to ^1^H *T*_2_ ≤ 30 ms) pointing to substantial IF figures, while STD < 0.15% (^1^H *T*_2_ > 50 ms) to negligible IF.

With the aim of broadening the scope of IF-STD, a polypropylene imine dendrimer of generation 4 (PPI-G4) was also analyzed (Figure 4). Despite its very low MW (3514 Da), STD_off-on_ signals were observed at 298 K that increased at 278 K. A topological analysis^25^ revealed that, independently of the on-saturation site, 0.06 ppm (close to the core H1 protons) or 4.16 ppm (close to peripheral H14), more intense STD signals were observed on going from the periphery to the core, in agreement with the known slower dynamics and lower *T*_2_ values at the dendritic core.^26,27^ Also, for the internal protons, larger STD factors were seen when saturating at 0.06 ppm. This experiment illustrates a fundamental property of IF-STD: the signal is generated within a single state as opposed to several states with different degrees of aggregation. According to the mechanism depicted in Figure 4A, magnetization in PPI-G4 transfers by spin diffusion intramolecularly from the saturated less mobile nuclei at the core (characterized by very short *T*_2_) to the more flexible domains at the periphery (larger *T*_2_).

**Figure 4:**
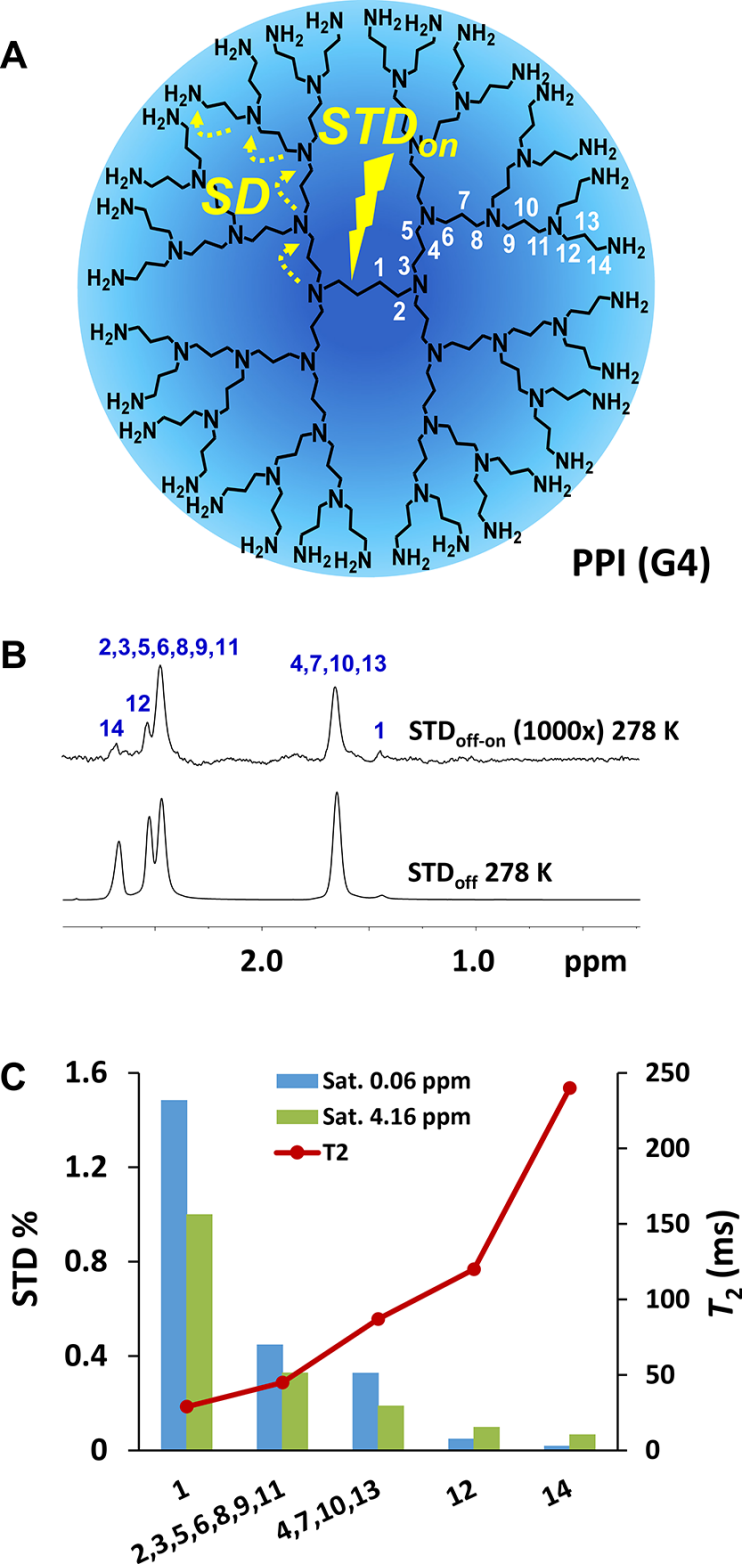
Structure of PPI-G4 and schematic representation of the IF-STD experiment (A). IF-STD spectrum of PPI-G4 (750 MHz, 278 K, 10 g/L in D_2_O, on-saturation 0.06 ppm) (B). STD factors saturating at 0.06 and 4.16 ppm (750 MHz, 278 K) and ^1^H *T*_2_ (500 MHz, 298 K) (C).

Contrary to a reductionist visible–invisible dichotomy, a continuous distribution of nuclei with diverse dynamics is envisioned in polymers (single state) so that *T*_2_, recorded in conventional NMR experiments, would stand for mean values of all nuclei in the NMR-visible fraction (regions H_I_ and H_II_ in Figure 1), while nuclei with lower *T*_2_ would constitute the IF (region H_III_ in Figure 1). To evaluate IF-STD for selectively tunning nuclei at the visible–invisible interphase, we compared ^1^H *T*_2_ values obtained via conventional Carr–Purcell–Meiboom–Gill (CPMG)^28,29^ pulse sequence (detection of all NMR-visible nuclei) and a modified IF-STD-CPMG selected to probe nuclei at the visible–invisible interphase. Figures 5 and S20 show, for the H2 of CS, superimposable signal decays with the CPMG time in the conventional CPMG and hybrid IF-STD_off_-CPMG spectra (*t*_sat_ 0.5–3 s), accounting for a ^1^H *T*_2_ of 25 ms. This result rules out potential heating artifacts in the experiment introduced by the saturation pulse. By contrast, the IF-STD_off-on_-CPMG experiment showed a steeper decay, in agreement with the expected slower dynamics of nuclei at the visible–invisible interphase (Figure 5). More pronounced decays were observed when reducing the *t*_sat_ from 3.0 to 0.5 s, affording ^1^H *T*_2_ values of 12 and 6 ms, respectively (same trend for H3–H6 in Figure S21). This result is consistent with the spin diffusion mechanism and the single state model depicted in Figure 1. The shorter the *t*_sat_, the shorter the distance for the propagation of the saturation from the invisible (H_III_) to the visible domain (eq 3), leading to STD_off-on_ spectra with a greater contribution of the H_II_ protons in closer proximity to the IF. So, IF-STD emerges as a tool for the signal-edition of nuclei at the visible–invisible interphase, a platform for gaining an insight into the IF.

**Figure 5:**
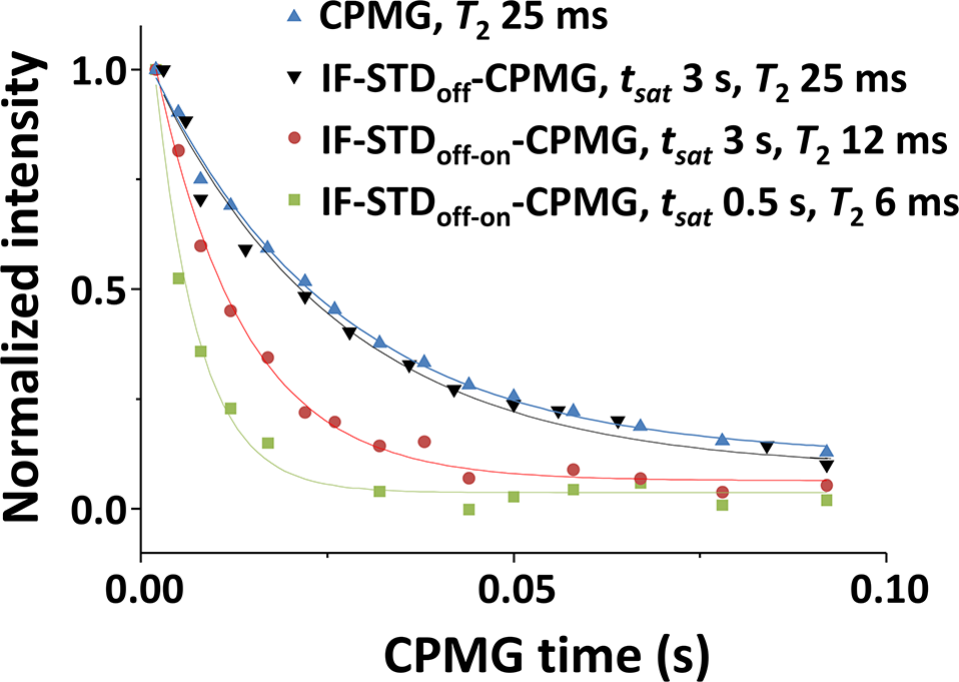
Effect of *t*_sat_ during IF-STD-CPMG on the ^1^H *T*_2_ of the visible–invisible interphase. Normalized ^1^H intensities (*I*/*I*_0_) for the H2 of CS (80 kDa, DA 14, 10 g/L in pD 4.5 acetate buffer) as a function of the CPMG time in conventional CPMG and hybrid IF-STD-CPMG (750 MHz, 298 K).

A similar strategy combining IF-STD and a diffusion encoding step based on the BPPSTE experiment (Bipolar Pulse Field Gradient-STimulated Echo)^30^ added further evidence for the single state model. The apparent translational diffusion coefficient of the CS fraction edited by the hybrid IF-STD-BPPSTE was compared with that obtained by the conventional BPPSTE. No difference among their Stejskal–Tanner plots was observed (several *t*_sat_ tested; Figure S22) in agreement with a single diffusion coefficient for all NMR-visible nuclei (regions H_I_ and H_II_ in Figure 1), independent of their spatial proximity to the IF.

In conclusion, IF-STD is described as a fast and reliable tool to unveil the IF of polymers in solution. The saturation of a polymer in a region of the NMR spectrum with IF (very short ^1^H *T*_2_) results in an efficient propagation of the magnetization by spin diffusion to a visible–invisible interphase with larger ^1^H *T*_2_ and faster dynamics (STD_on_). Visualization of this interphase in a difference STD_off-on_ spectrum has revealed IF more common than previously thought, with relevant IF figures for a wide variety of natural and synthetic polymers when STD > 0.4% at 750 MHz. This is the case for charged rigid polysaccharides like hyaluronic acid, carrageenan and CS, weak polyelectrolytes (PAA, PMAA, PPI dendrimer) and even uncharged polymers like PVP. In agreement with the spin diffusion mechanism, larger STD factors revealed for the larger MW and higher concentrations (aggregation and viscosity), lower temperatures, and lower magnetic fields. More intense STD were also obtained the closer the on-saturation to the polymer visible signals, and the larger the *t*_sat_ and BW.

A fundamental property of the IF-STD experiment is that the signal is generated within a single state comprising polymer domains with different dynamics. As nuclei edited by IF-STD at the visible–invisible interphase are in close spatial proximity to the IF (tunable with *t*_sat_), they represent a convenient platform from which gaining an insight into the IF itself. For instance, combining IF-STD with a CPMG sequence has allowed for CS to determine *T*_2_ values for the visible–invisible interphase 4-fold lower than the average *T*_2_ probed by conventional CPMG for all visible nuclei. This result confirms that contrary to a reductionist visible–invisible dichotomy in polymers, there is a continuous distribution of nuclei with diverse dynamics in agreement with the proposed single state mechanism.

## Supporting Information Available

Materials, NMR methods, IF-STD spectra, and figures (PDF)
