# Supplementary material for: Unveiling an NMR-Invisible Fraction of Polymers in Solution by Saturation Transfer Difference
Source: ACS Macro Lett. 2021 Nov 10;10(12):1474–9. doi: 10.1021/acsmacrolett.1c00628 (PMC8697556; doi:10.1021/acsmacrolett.1c00628)
Supplement: Supplementary file 1 — mz1c00628_si_001.pdf [file mz1c00628_si_001.pdf]

Supporting Information for:

Unveiling an NMR-Invisible Fraction of Polymers in Solution by Saturation Transfer Difference

Ramon Novoa-Carballal,[a] Manuel Martin-Pastor,[b] and Eduardo Fernandez-Megia[a]*

[a] Centro Singular de Investigación en Química Biolóxica e Materiais Moleculares (CIQUS), Departamento de Química Orgánica, Universidade de Santiago de Compostela, Jenaro de la Fuente s/n, 15782 Santiago de Compostela, Spain.

[b] Unidade de Resonancia Magnética, Área de Infraestructuras de Investigación, CACTUS, Universidade of Santiago de Compostela, 15782 Santiago de Compostela, Spain.

Table of Contents

1. Materials S3

2. Nuclear Magnetic Resonance S4

3. IF-STD Spectra and Figures S15

4. References S32

1. Materials

Chitosan·HCl (CS) was obtained from FMC BioPolymer as hydrochloride salt [Protasan Cl 113, batch number FP-110-02; M_w 80 kDa, PDI 1.48 by SEC-MALLS; degree of acetylation (DA) 14% by ^{1}H NMR].[1] Carrageenan (M_n 647 kDa, PDI 1.61, by SEC-MALLS) was obtained from CEAMSA and ulvan (M_n 524 kDa by SEC-MALLS) extracted from green algae. Poly(propylene imine) dendrimer of G4 (PPI-G4) with batch number 32Am-12cc-C01 was obtained from SyMO-Chem. Pullulan samples were purchased from Polymer Standards Service (M_n 11.8 kDa, PDI 1.10; M_n 112 kDa, PDI 1.12; M_n 788 kDa, PDI 1.23). Poly(vinyl alcohol) (PVA) was purchased from Alfa Aesar (M_w 306 kDa, PDI 1.13 by SEC-MALLS, batch number B08R009). All other polymers were obtained from Sigma-Aldrich-Fluka: poly-L-lysine·HBr (PLL, M_n 45 kDa, from Sigma, batch number 120M5014V), polyvinylpyrrolidone (PVP, average molecular weight 360 kDa, from Aldrich, batch number 1300807), poly(acrylic acid) (PAA, M_v ~ 450 kDa, from Aldrich, batch number U12990), hyaluronic acid (M_w 160 kDa by SEC-MALLS, from Aldrich, batch number 057k3789), poly-L-glutamic acid sodium salt (PGA, M_v 14 kDa and 35 kDa, from Fluka, batch numbers 1255079 and 017k5108v, respectively), branched polyethyleneimine (PEI-branched, M_w ~ 25 kDa by LS, from Aldrich, batch number 02816BD), poly(ethylene glycol) (PEG, M_n 10 kDa, from Aldrich, batch number 11180 231041899), polyacrylamide (M_v 5000 kDa, from Aldrich, batch number 1285399), poly(styrenesulfonate) (PSS, M_v 1000 kDa, from Aldrich, batch number 10805KC), and sodium poly(methacrylate) (PMAA, M_v 30 kDa, PDI < 1.2, from Aldrich, batch number 1405491).

Depolymerization of CS. Commercial CS (Protasan Cl 113) was depolymerized by addition of nitrous acid.[2] By adjusting the amount of $NaNO_2$ added and fractionating by precipitation with

EtOH or ultrafiltration (Amicon YM1), CS samples of lower MW were obtained: M_n 2.3 kDa, PDI 1.15; M_n 5.9 kDa, PDI 1.15; M_n 29 kDa, PDI 1.19 by SEC-MALLS.

2. Nuclear Magnetic Resonance

CS was dissolved in a pD 4.5 buffer solution consisting of 350 mM CD_3CO_2D / 135 mM NaOD in D_2O. All other polymer solutions were prepared in D_2O, unless noted. NMR spectra were acquired on an Agilent Inova or Bruker NEO spectrometers operating at 17.6 T (750 MHz resonance of proton), Bruker DRX-500 (operating at 11.74 T, 500 MHz resonance of proton), Varian Inova 400 (operating at 9.39 T, 400 MHz resonance of proton) or Bruker DPX-250 (operating at 5.87 T, 250 MHz resonance of proton) spectrometers. Spectra were processed and analyzed with Mnova software (Mestrelab Research S.L.).

Determination of NMR Invisible Fractions (IF). The percentages of NMR IF shown in Table 1 were determined at 750 MHz. To this end, the integral of the ^{1}H NMR signals indicated in Table S1 was compared to that of an external reference (maleic acid or trimethylsilyl propionic acid placed in a coaxial capillary inside the NMR tube) in a series of quantitative ^{1}H NMR spectra recorded at increasing temperatures (278, 298, 313, 328, and 343 K). The IF% at any T is defined as shown in Eq S1. IF figures lower than 1.5% were considered as zero.

$$IF\% = \frac{\text{Integral at 343 K} - \text{Integral at a lower T}}{\text{Integral at 343 K}} \text{ x } 100 \qquad \text{Eq S1}$$

^{1}H NMR Data of the Polymers under Study (750 MHz, D_2O, 298 K). CS: δ 2.07 (s, Ac), 3.17 (br s, H2 GlcN), 3.35-4.42 (m, H2 GlcNAc, H3-H6). PLL: δ 1.40-1.59 (m, 2H, H4), 1.71-1.93 (m, 4H, H3, H5), 3.07 (t, J = 7.54 Hz, 2H, H6), 4.24-4.45 (m, 1H, H2). PVP: δ 1.50-1.89 (m, 2H, H1), 1.99-2.15 (m, 2H, H4), 2.27-2.63 (m, 2H, H5), 3.12-3.44 (m, 2H, H3), 3.55-3.99 (m, 1H, H2). PAA: δ 1.52-2.12 (m, 2H, H2), 2.32-2.63 (m, 1H, H1). Hyaluronic acid: δ 2.04 (s, 3H, Ac), 3.22-4.15 (m, 10H, H2-H6 GlcA and GlcNAc), 4.47 (br s, 1H, H1 GlcA), 4.56 (br s, 1H, H1 GlcNAc). PGA: δ 1.90-2.14 (m, 2H, H2), 2.20-2.36 (m, 2H, H3), 4.34 (dd, J = 8.9, 5.4 Hz, 1H, H1). Pullulan: δ 3.48 (t, J = 9.3 Hz, 1H), 3.58-3.66 (m, 4H), 3.70 (q, J = 9.5 Hz, 2H), 3.80 (d, J = 9.3 Hz, 1H), 3.90 (d, J = 8.1 Hz, 1H), 3.92-3.99 (m, 5H), 4.03 (t, J = 9.3 Hz, 1H), 4.97 (d, J = 3.1 Hz, 1H), 5.39 (dd, J = 18.3, 3.4 Hz, 1H). Carrageenan: δ 3.20-4.75 (m, 6H), 5.10 (s, 1H, H1-*k*), 5.29 (s, 1H, H1-*i*), 5.40 (s, 1H, H1-L). PSS: δ 0.50-2.33 (m, 3H, H1, H2), 5.96-6.96 (m, 2H, H3), 7.20-8.00 (m, 2H, H4). PMAA: δ 0.75-1.45 (m, 3H, H2), 1.48-2.25 (m, 2H, H1). Polyacrylamide: δ 1.40-1.90 (m, 2H, H2), 2.10-2.50 (m, 1H, H1). PGA: δ 1.81-2.10 (m, 2H, H2), 2.20-2.42 (m, 2H, H3), 4.30-4.45 (m, 1H, H1). PEG: δ 3.50-3.88 (m, CH_2O). PEI-branched: δ 0.97-3.75 (m, CH_2N). PPI-G4: δ 1.43-1.53 (m, H1), 1.61-1.75 (m, H4,7,10,13), 2.45-2.53 (m, H2,3,5,6,8,9,11), 2.54-2.59 (m, H12), 2.63-2.73 (m, H14). Ulvan: δ 1.25-1.36 (m, 3H, CH_3) 3.20-4.90 (m, 11H, H1-H6). PVA: δ 1.36-2.20 (m, 2H, H2), 3.50-4.15 (m, 1H, H1).

Invisible Fraction-Saturation Transfer Difference (IF-STD). IF-STD experiments were acquired at 750 MHz. The number of scans was 96, except for CS samples (128) and PPI-G4 (256). Unless otherwise mentioned, the inter-scan delay (d_1) was 6 s immediately followed by a saturation time of 3 s. The on- and off-saturation consisted of a train of low power saturation pulses of gaussian shape with nominal duration of 35.4 ms and separated by a 1 ms delay. The effective band-width of the saturation applied (BW^{eff}) was 130 Hz, a value that was determined experimentally in the spectrometer with a sucrose sample following the method described below. The frequency of the on-saturation was placed in an empty region of the ^1H spectrum, at a frequency -1125 Hz from the right end (starting of noise) of the lowest ppm visible signal in the NMR spectrum of each polymer. This frequency is equivalent to -1.5 ppm in our spectrometer operating at 750 MHz. The frequency of the off-saturation was placed at 20 ppm, a position that is more than 10000 Hz away from any visible signal in all the ^1H spectra analyzed. $STD_{off\text{-}on}$ spectra were obtained with the scans corresponding to the STD_{on} and STD_{off} experiments interleaved during the acquisition and the corresponding FIDs subtracted automatically by the phase cycle. STD_{off} control spectra were acquired by application of the off-saturation pulse train only, without any FID subtraction. STD factors were calculated from the integrals of the $STD_{off\text{-}on}$ and STD_{off} spectra using equation S2.

$$STD\% = \frac{\mathrm{STD_{off}} - \mathrm{STD_{on}}}{\mathrm{STD_{off}}} \text{ x } 100 \qquad \text{Eq S2}$$

For this purpose, the two spectra were processed identically. Integration ranges of the polymer signals are shown in Table S1. STD factors lower than 0.05% were considered as zero.

Table S1. Integration range of the polymer ^{1}H signals selected for IF-STD analysis and location of the on-saturation pulse.

Polymer	Integration range (ppm)	On-saturation
Hyaluronic acid	1.70-2.10	0.36
Carrageenan	3.40-3.85	1.92
PSS	0.05-2.20	-1.00
Chitosan (CS)	1.70-2.10	0.54
PVP	1.39-1.92	-0.08
PMAA	0.50-1.40	-0.62
Ulvan	1.20-1.40	-0.35
PAA	1.00-1.95	0.03
Polyacrylamide	1.00-2.70	-0.10
Pullulan	3.25-4.00	1.85
PVA	1.00-2.00	0.00
PGA	1.50-2.50	0.38
PLL	0.70-1.70	-0.22
PEG	3.20-3.80	2.00
PEI-branched	2.20-2.90	0.22
PPI-G4	1.40-1.50 (H1)<br>1.55-1.82 (H4,7,10,13)<br>2.40-2.48 (H2,3,5,6,8,9,11)<br>2.48-2.57 (H12)<br>2.57-2.67 (H14)	0.06 and 4.16

Experimental Determination of the Effective Band-width (BW^{eff}) Covered by the Saturation in an IF-STD Experiment. BW^{eff} (in Hz) depends on several experimental parameters, including the shape of the pulse, its nominal duration and power level, the inter-pulse delay used in the saturation train as well as the spectrometer hardware (RF amplifier and probe). In this section, a method is proposed for measuring BW^{eff} in any modern spectrometer (year 2000 and beyond). The method measures STD_{off-on} and STD_{off} experiments of a sample of 50 mM sucrose in D_2O under analogue conditions to those described above for the measurement of the IF-STD spectra of polymers, except for those parameters mentioned below. The number of scans was 64. The on-saturation was placed at 3.737 ppm, corresponding to the overlapping signals of two methylene protons that are assigned to H6g and H6f of sucrose. Fourteen pairs of STD spectra (STD_{off-on} and STD_{off}) were measured at a series of pulse-lengths for the shaped gaussian pulse used for the saturation. The gaussian pulse is repeated during the saturation train to cover the same total saturation time of 1 s in each experiment. The nominal durations explored for the gaussian pulse were 14.1, 15.1, 16.3, 17.7, 19.3, 21.2, 26.5, 28.3, 30.3, 32.6, 35.4, 38.6, 42.4, and 50 ms. In each case, the RF power of the gaussian pulse was set to produce the same tilt angle of 90 º. This power was calculated based on the reference of the hard 90 º proton pulse by using the *ShapeTool/Pandora's box* module included in the TopSpin/VNMRJ control software. The same software was used to calculate the theoretical excitation band-width in Hz covered by the gaussian shaped pulse (BW^{calc}). The two mentioned calculations assume a linear response in the power of the RF proton amplifier of the spectrometer, a requirement fulfilled by modern spectrometers equipped with linear RF amplifiers, which can be even improved by the experimental correction of the linearization that is done during the spectrometer's installation.

For small molecules in solution, as sucrose in D_2O, at the typical frequencies of the NMR spectrometers, the subtracted STD_{off-on} spectrum shows the highest intensity for the peak/s that

is/are being affected by the saturation. In the same spectrum, the presence of NOE peaks that denote proton-proton proximity are of very low intensity (a few percent of the saturated peak/s) and can be easily identified as they appear with opposite sign respect to the peak/s that is/are being saturated. Other possible peaks are those generated by spin-diffusion phenomena (three spin effects), which may have the same sign that the saturated peak/s but their intensity is very modest and comparable to that of the NOE. The remaining peaks are those that are neither affected by the saturation, NOE or spin-diffusion; they will appear with null intensity in the $STD_{off\text{-}on}$ spectrum. Under these premises, it is straightforward to distinguish in a $STD_{off\text{-}on}$ spectrum of sucrose the peak/s that is/are being affected by the on-saturation, and to determine experimentally an upper and lower limit values of BW^{eff} by measuring the distance in Hz from the central frequency position at which the on-saturation is applied to (a) the furthest peak identified as saturated (lower limit, $BW^{eff\_lowerlimit}$) and (b) the closest peak identified as not saturated (upper limit, $BW^{eff\_upperlimit}$). The lower and upper limits of BW^{eff} were measured for sucrose using the above series of gaussian saturation pulse-lengths in a Bruker NEO 750 MHz spectrometer (Figure S1). The results obtained are given in Table S2. Figure S2 represents BW^{calc}, this is the excitation band-width calculated with the spectrometer software, and the experimentally determined $BW^{eff\_lowerlimit}$ and $BW^{eff\_upperlimit}$ at each pulse-length. As a linear relationship can be expected between BW^{calc} and BW^{eff}, the points were fitted to a straight line so that at each pulse-length, the value of BW^{eff} passes in between $BW^{eff\_lowerlimit}$ and $BW^{eff\_upperlimit}$. Such a linear dependence provides BW^{eff_fit} that is equivalent to BW^{eff} in the IF-STD experiment (Figure S2 and Table S2). According to these results, under our experimental conditions, the BW^{eff} at each pulse-length corresponds to twice the value of that calculated by BW^{calc} in the Bruker NEO 750 MHz spectrometer. Under identical experimental conditions, a BW^{calc} equal to BW^{eff} was obtained for the Varian INOVA 750 MHz spectrometer.

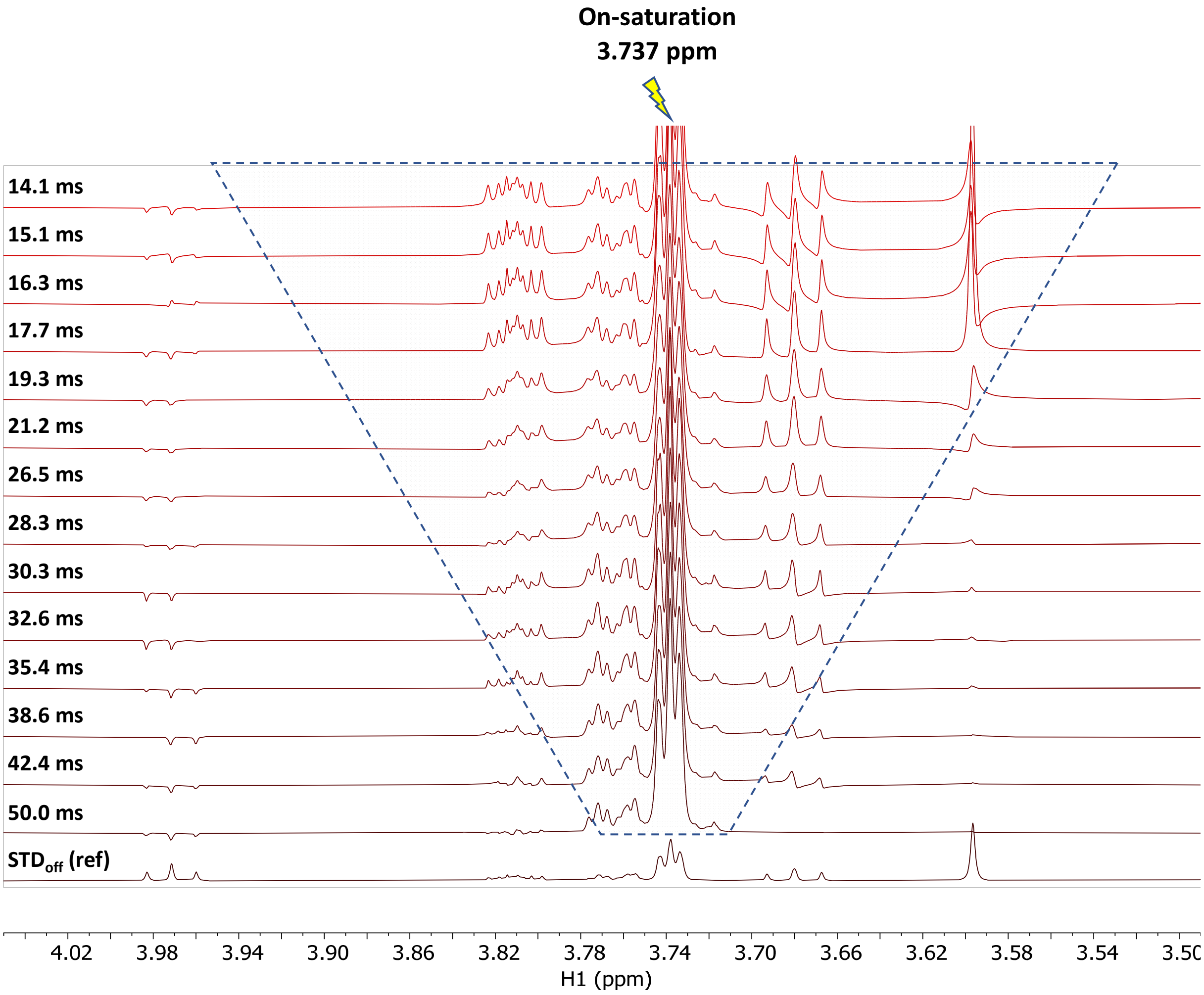

Figure S1. Stack of $STD_{off\text{-}on}$ spectra of sucrose (50 mM in D_2O) recorded for the determination of the experimental effective band-width (BW^{eff}) for the IF-STD experiment in our Bruker NEO 750 MHz spectrometer. The nominal duration of the gaussian saturation pulses (in ms) is indicated. The position of the on-saturation at 3.737 ppm is shown by an arrow. At the bottom is the STD_{off} spectrum (reference). In each $STD_{off\text{-}on}$ spectrum the distance between the pair of stripped lines (in Hz) corresponds to the BW^{eff_fit} shown in Table S2.

Table S2. Determination of the experimental effective band-width (BW^{eff}) of the IF-STD experiment in our Bruker NEO 750 MHz spectrometer. $BW^{eff\_lowerlimit}$ and $BW^{eff\_upperlimit}$ were determined experimentally in $STD_{off\text{-}on}$ spectra of sucrose (50 mM in D_2O) recorded with a series of gaussian saturation pulse-lengths. BW^{calc} is the excitation band-width calculated with the Bruker Shapetool module of TopSpin at each gaussian-pulse length. $BW^{eff\text{-}fit}$ is the value obtained by fitting the points of Figure S2 to a straight line.

**Gaussian pulse length [(a)] (ms)**	**BW^{calc} (Hz)**	**$BW^{eff\_lowerlimit}$ (Hz)**	**$BW^{eff\_upperlimit}$ (Hz)**	**BW^{eff_fit} (Hz)**
50	42.44	62	88	84.88
42.4	50	114	128	100
38.6	55	114	128	110
35.4	60	112	220	120
32.6	65	112	220	130
30.3	70	112	220	140
28.3	75	112	220	150
26.5	80	112	220	160
21.2	100	112	220	200
19.3	110	112	220	220
17.7	120	218	330	240
16.3	130	218	330	260
15.1	140	218	330	280
14.1	150	218	330	300

[(a)] Nominal duration of the gaussian pulses used for the saturation. The RF power of the pulses was calibrated to produce a tilt angle of 90 º.

Average $BW^{eff} = (BW^{eff\_upperlimit} + BW^{eff\_lowerlimit})/2$

Linear fit : $BW^{eff\_fit} = 2.01 \pm 0.08 \cdot BW^{calc}$

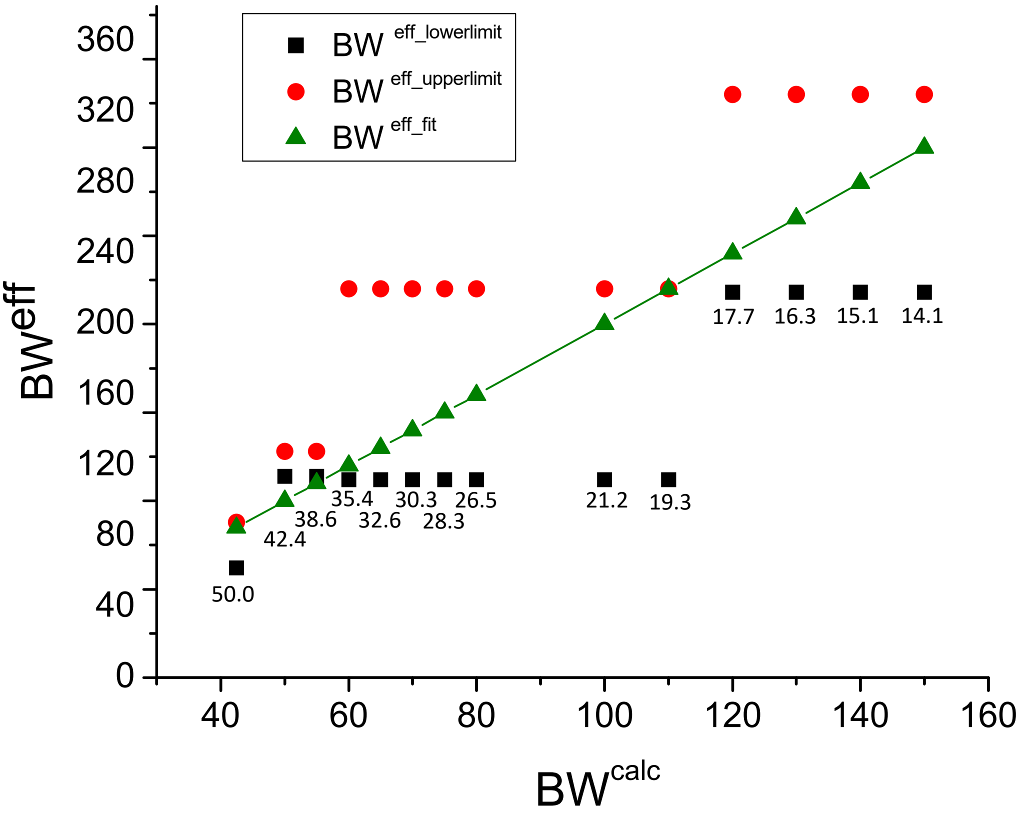

Figure S2. Plot of BW^{calc} respect to the measured $BW^{eff\_lowerlimit}$ (black squares) and $BW^{eff\_upperlimit}$ (red circles) for sucrose (50 mM in D_2O) in our Bruker NEO 750 MHz spectrometer. At each value of BW^{calc} the numbers indicate the corresponding pulse-length of the gaussian pulse used in ms. As there is a linear relationship between BW^{calc} and BW^{eff}, the points were fitted to a straight line that passes in between $BW^{eff\_lowerlimit}$ and $BW^{eff\_upperlimit}$ at each pulse-length (green triangles). Under these conditions the fitting equation obtained is: BW^{eff_fit} (Hz) = $2.01 \pm 0.08 \cdot BW^{calc}$. The fitting provides BW^{eff_fit} which corresponds to the actual BW^{eff} in IF-STD experiments.

Determination of ¹H T_2 Relaxation Times. ¹H T_2 values were determined at 500 MHz using the Carr-Purcell-Meiboom-Gill (CPMG)[3] pulse sequence $[90^0x-(\tau - 180^0y - \tau)_n$, where 2τ is a fixed echo time ($\tau = 0.7$ ms), n is the number of echoes, and the total CPMG duration (t) is given by $2\tau \cdot n$. The CPMG duration (t) was linearly varied along 16 steps between a minimum value of 1.4 ms ($n = 1$) and a maximum of ca. 6 to 7 times the highest T_2. At each step, a spectrum was acquired with 64 scans. The interscan relaxation delay (d_1) was larger than 5 times the highest ¹H T_1 in the sample. The absolute signal integral (I) at each value of t was fitted to the mono-exponential equation S3 to determine the relaxation time T_2:

$$I(t)/I_0 = \exp\left(-t/T_2\right) \qquad \text{Eq S3}$$

where $I(t)$ and I_0 are the observed signal integrals at a certain value of t and for t equal to the minimum value of the series (t =1.4 ms), respectively. OriginPro 9.0 Software (Originlab Corporation) was used to perform the exponential fittings to obtain the ¹H relaxation times T_2.

Determination of ¹H T_2 values at the *visible-invisible interphase* of polymers was performed by means of an IF-STD–CPMG experiment. This hybrid pulse sequence was built with a T_2 filter based on the conventional CPMG sequence.[3] The experimental conditions used for STD_{off}–CPMG and STD_{off-on}–CPMG experiments were identical to those described above for the CPMG and IF-STD experiments, using 64 scans.

Determination of Translational Diffusion Coefficients. Translational diffusion coefficients (D) were measured at 750 MHz with the BPPSTE experiment based on the stimulated echo and bipolar pulse field gradients (PFG) (sequences Doneshot in the Agilent and ledbpgp2s in the Bruker pulse sequence libraries, respectively).[4] The PFG strength was calibrated with a reference sample of D_2O 99.9% at 298 K, D= $1.87 \cdot 10^{-9}$ m^2s^{-1}. For the measurements with polymers, the diffusion delay was

set to 3 s, the duration δ of the PFG encoding diffusion was set to 4 ms. The strength of the PFG was linearly varied between 4 and 58 $G \cdot cm^{-1}$ along 20 steps, each one with acquisition of an FID. Diffusion coefficients were determined by analysis of the signal integrals along the 20 spectra and non-linear fitting to the Stejskal-Tanner equation governing the integral attenuation in the experiment.

Determination of diffusion values was also performed with an IF-STD-BPPSTE experiment that used identical parameters. The later pulse sequence was built by incorporating the IF-STD scheme and the parameters described above to the conventional BPPSTE pulse sequence.

3. IF-STD Spectra and Figures

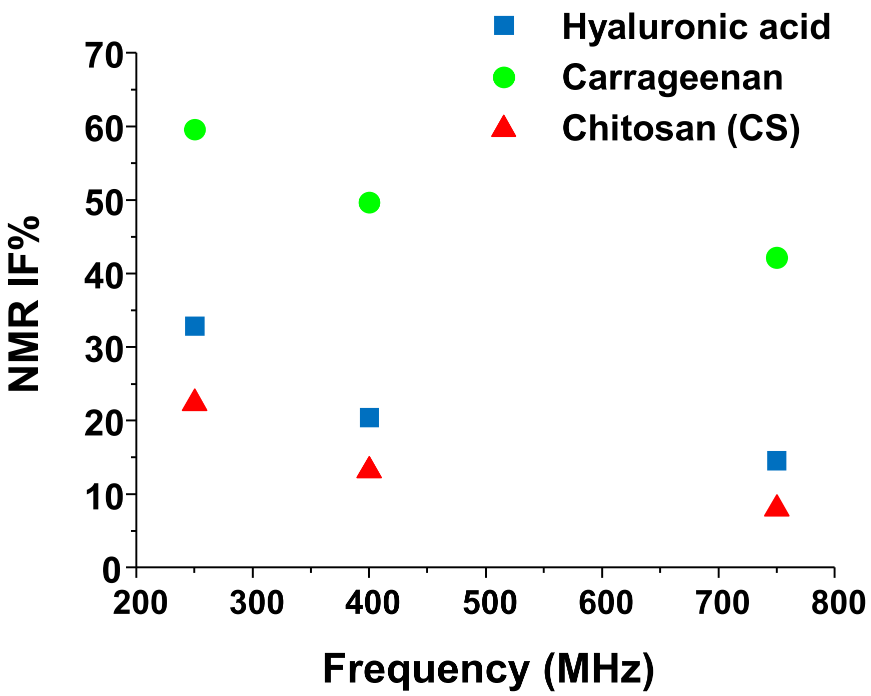

Figure S3. ^{1}H NMR IF of CS, hyaluronic acid and carrageenan at three magnetic fields (250, 400 and 750 MHz). Hyaluronic acid and carrageenan dissolved in D_2O (10 mg/mL), CS in 350 mM CD_3CO_2D/135mM NaOD in D_2O (10 mg/mL).

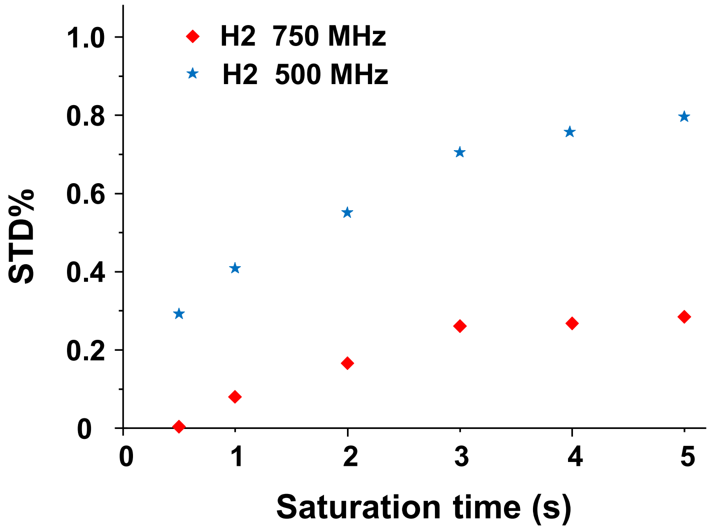

Figure S4. STD factor as a function of the saturation time (on-saturation at 0.54 ppm) and magnetic field (500 and 750 MHz) for the H2 of CS (80 kDa, DA 14) dissolved at 10 mg/mL in 350 mM CD_3CO_2D/135mM NaOD in D_2O.

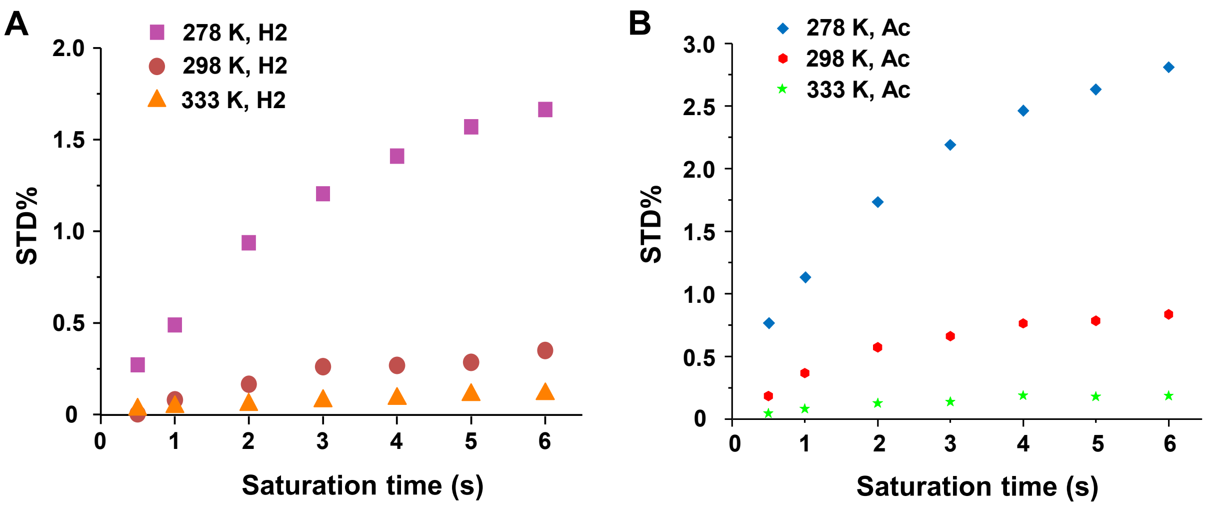

Figure S5. STD factor as a function of the saturation time (on-saturation at 0.54 ppm) and temperature (278, 298 and 333 K) for the H2 (A) and acetyl (B) of CS (80 kDa, DA 14) dissolved at 10 mg/mL in 350 mM CD_3CO_2D/135mM NaOD in D_2O.

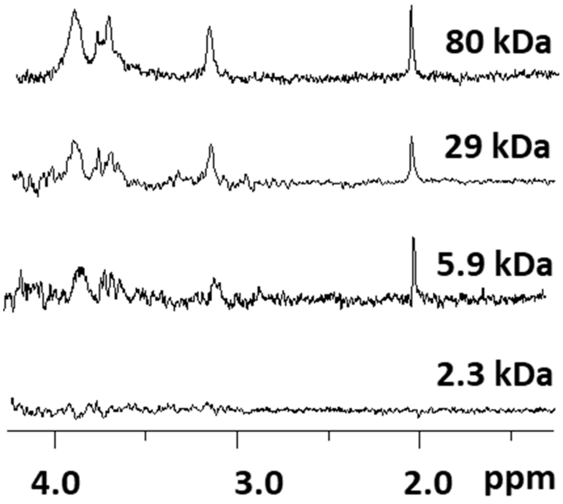

Figure S6. Effect of the MW of CS on the IF-STD intensity: STD_{off-on} spectra (750 MHz, 298 K, on-saturation at 0.54 ppm, saturation time 3 s) of CS (2.3-80 kDa, 10 mg/mL in pD 4.5 acetate buffer, D_2O). Vertical scale of STD_{off-on} spectra is shown 100×.

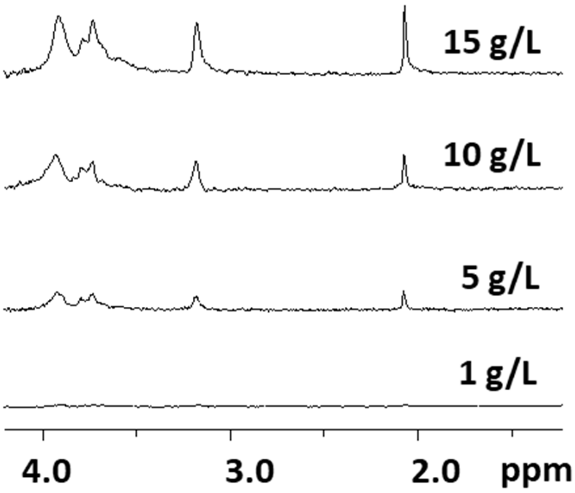

Figure S7. Effect of the concentration of CS on the IF-STD intensity: STD_{off-on} spectra (750 MHz, 298 K, on-saturation at 0.54 ppm, saturation time 3 s) of CS (80 kDa, 1-15 mg/mL in pD 4.5 acetate buffer, D_2O). Vertical scale of STD_{off-on} spectra is shown 100×.

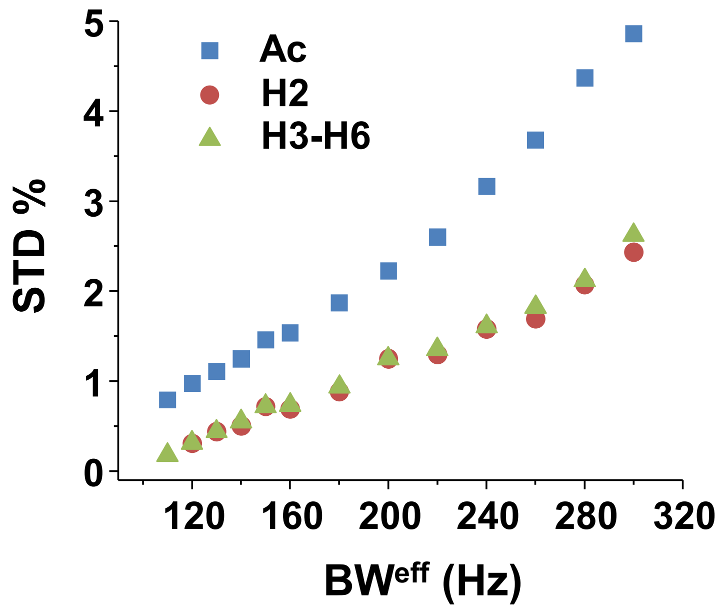

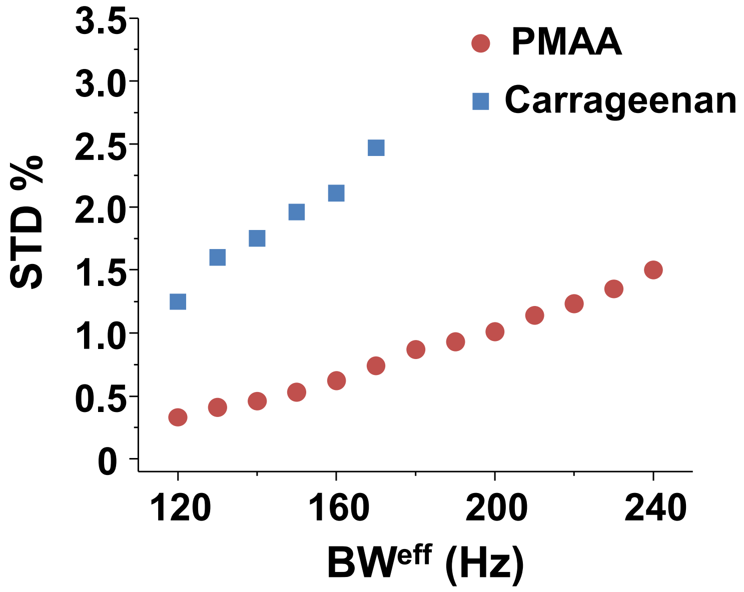

Figure S8. STD factors (750 MHz, 298 K, saturation time 3 s) as a function of the effective band-width (BW^{eff}) for CS (80 kDa, DA 14, 10 mg/mL in D_2O, on-saturation at 0.54 ppm), PMAA (30 kDa, 10 mg/mL in D_2O, on-saturation at -0.62 ppm), and carrageenan (647 kDa, 10 mg/mL in D_2O, on-saturation at 1.92 ppm).

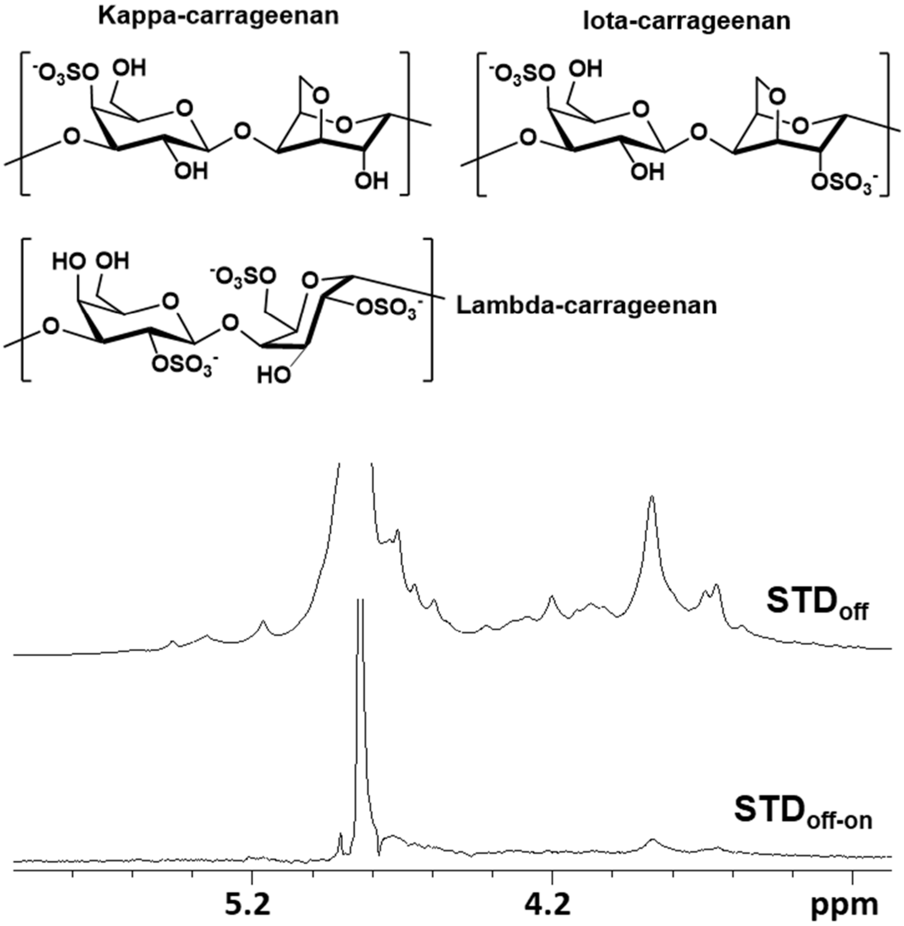

Figure S9. IF-STD spectra (750 MHz, 298 K, on-saturation at 1.92 ppm, saturation time 3 s) of carrageenan (647 kDa, 10 mg/mL in D_2O). Vertical scale of STD_{off-on} spectrum is shown 10×.

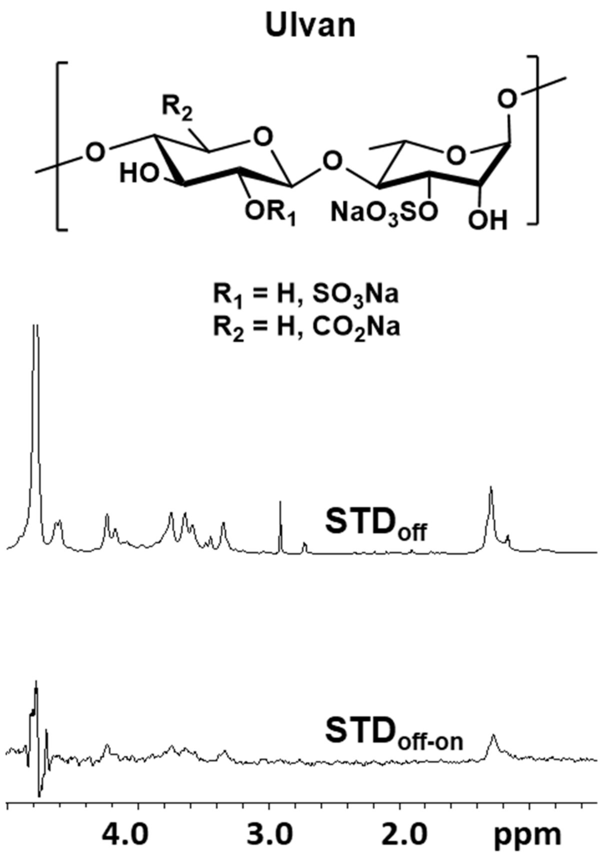

Figure S10. IF-STD spectra (750 MHz, 298 K, on-saturation at -0.35 ppm, saturation time 3 s) of ulvan (524 kDa, 10 mg/mL in D_2O). Vertical scale of $STD_{off\text{-}on}$ spectrum is shown 100×.

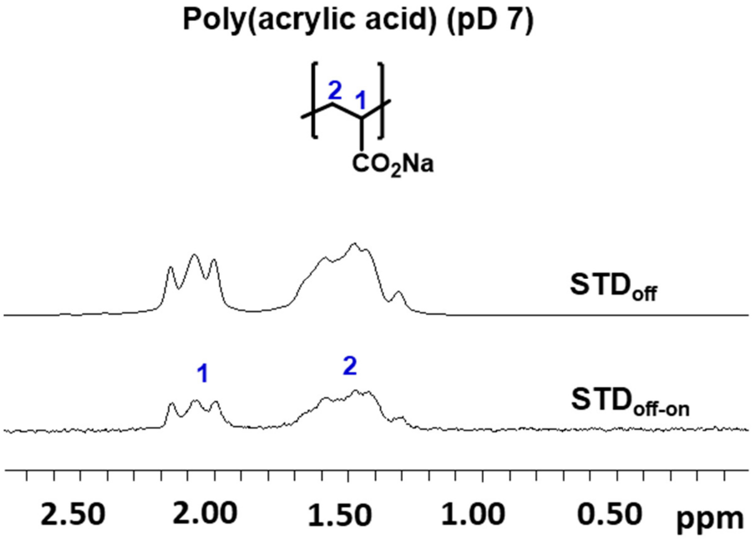

Figure S11. IF-STD spectra (750 MHz, 298 K, on-saturation at 0.03 ppm, saturation time 3 s) of poly(acrylic acid) (PAA 450 kDa, 10 mg/mL in D_2O, pD 7). Vertical scale of $STD_{off\text{-}on}$ spectrum is shown 10×.

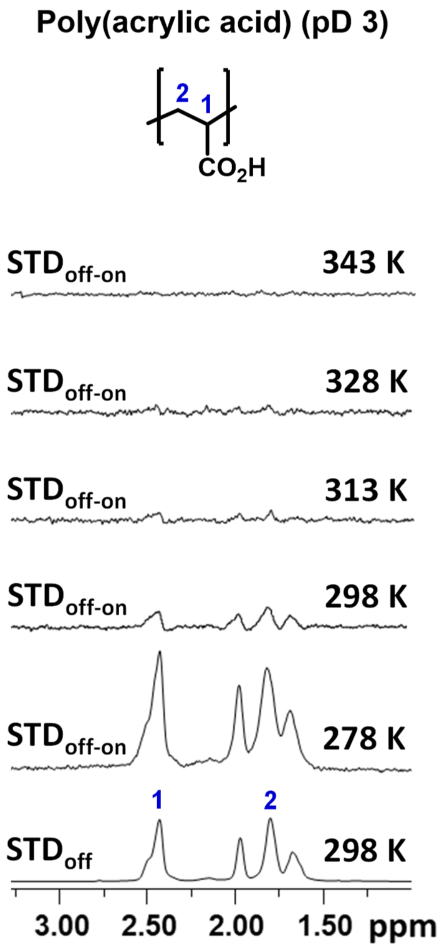

Figure S12. Effect of the temperature on the STD intensity: IF-STD spectra (750 MHz, 278-343 K, on-saturation at 0.03 ppm, saturation time 3 s) of poly(acrylic acid) (PAA 450 kDa, 10 mg/mL in D_2O, pD 3). Vertical scale of $STD_{off\text{-}on}$ spectra is shown 200×.

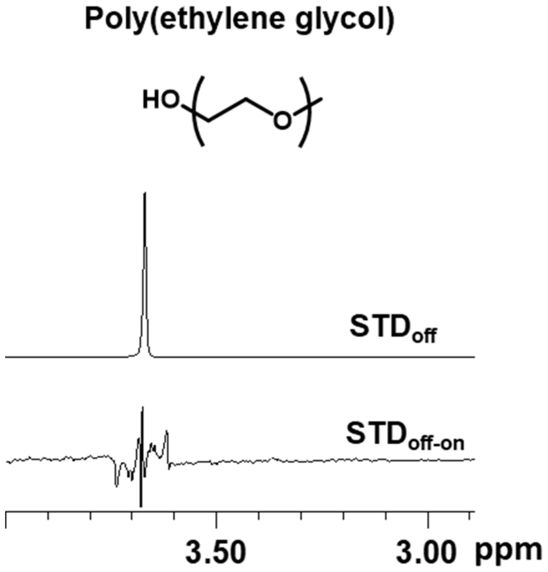

Figure S13. IF-STD spectra (750 MHz, 298 K, on-saturation at 2.00 ppm, saturation time 3 s) of poly(ethylene glycol) (PEG 10 kDa, 10 mg/mL in D_2O). Vertical scale of $STD_{off\text{-}on}$ spectrum is shown 15×.

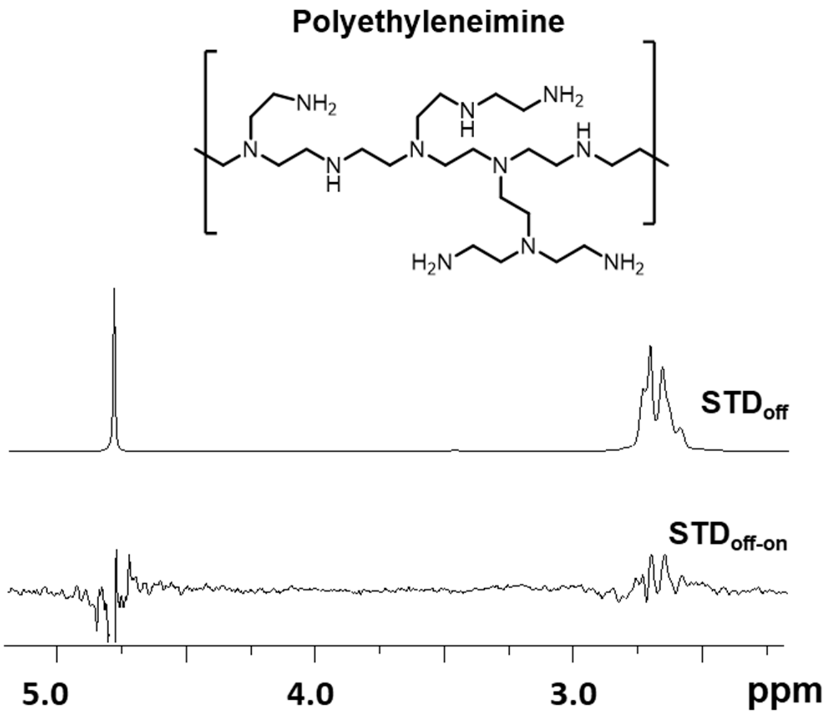

Figure S14. IF-STD spectra (750 MHz, 298 K, on-saturation at 0.22 ppm, saturation time 3 s) of branched polyethyleneimine (PEI-branched 25 kDa, 10 mg/mL in D_2O). Vertical scale of $STD_{off\text{-}on}$ spectrum is shown 100×.

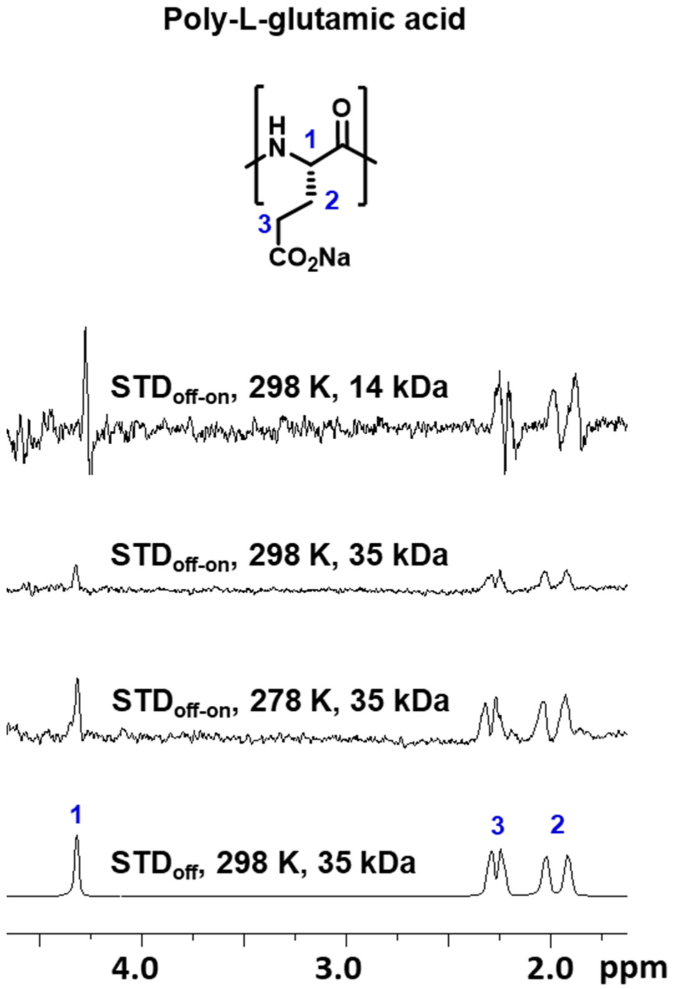

Figure S15. Effect of the MW and temperature on the STD intensity: IF-STD spectra (750 MHz, 278 and 298 K, on-saturation at 0.38 ppm, saturation time 3 s) of poly-L-glutamic acid sodium salt (PGA 14 and 35 kDa, 10 mg/mL in D_2O). Vertical scale of $STD_{off\text{-}on}$ spectra is shown 300×.

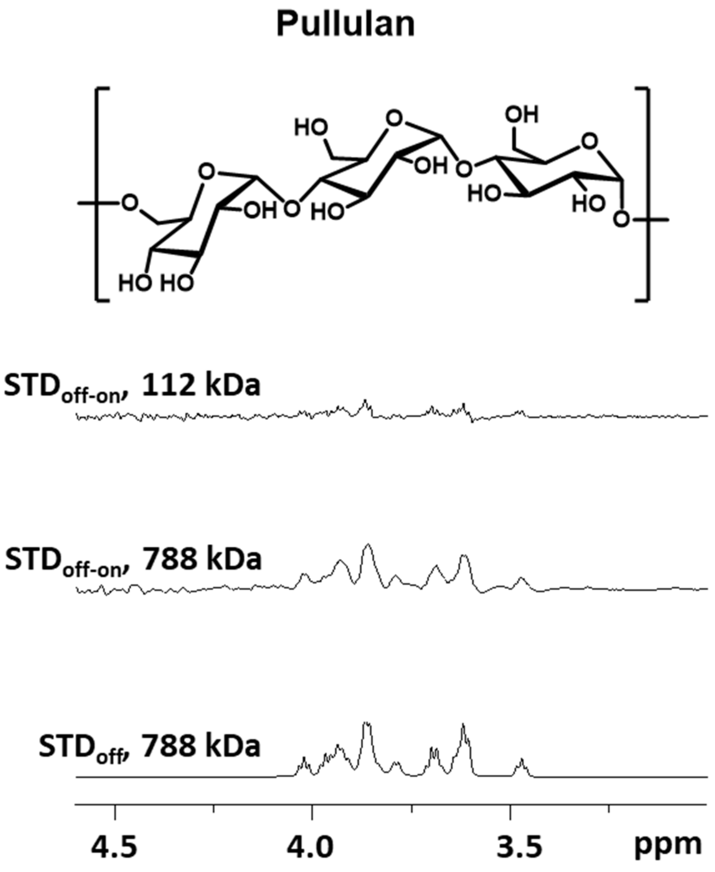

Figure S16. Effect of the MW on the STD intensity: IF-STD spectra (750 MHz, 298 K, on-saturation at 1.85 ppm, saturation time 3 s) of pullulan (112 and 788 kDa, 10 mg/mL in D_2O). Vertical scale of $STD_{off\text{-}on}$ spectra is shown 100×.

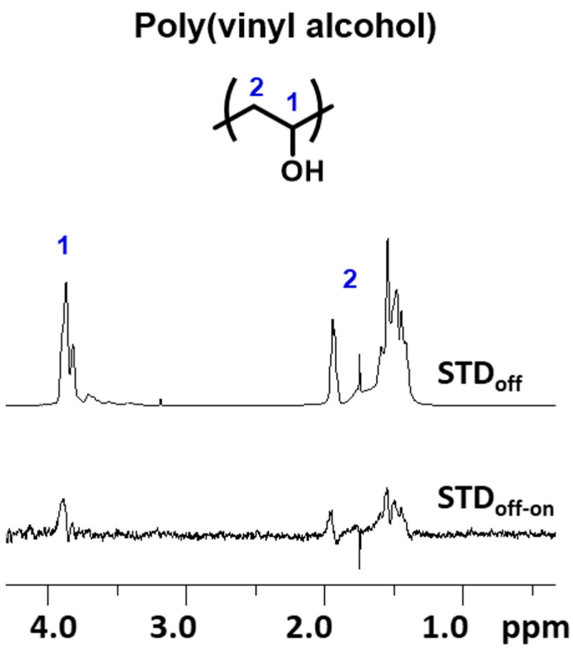

Figure S17. IF-STD spectra (750 MHz, 298 K, on-saturation at 0.00 ppm, saturation time 3 s) of poly(vinyl alcohol) (PVA 306 kDa, 10 mg/mL in D_2O). Vertical scale of $STD_{off\text{-}on}$ spectrum is shown 100×.

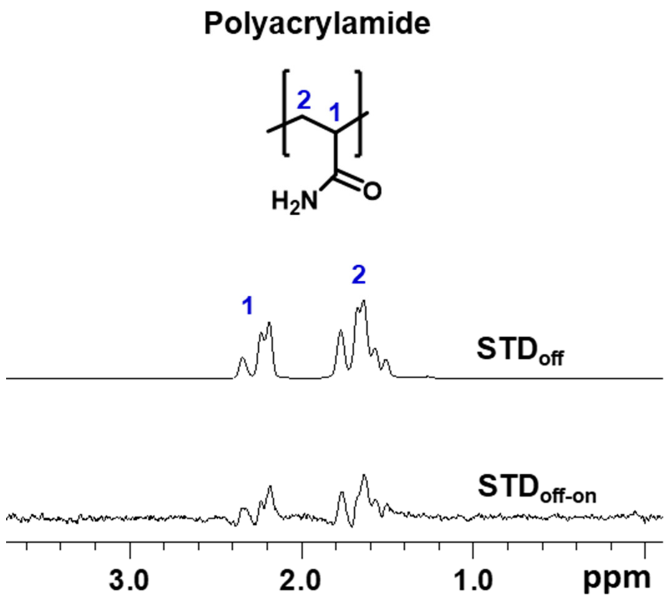

Figure S18. IF-STD spectra (750 MHz, 298 K, on-saturation at -0.10 ppm, saturation time 3 s) of polyacrylamide (5000 kDa, 10 mg/mL in D_2O). Vertical scale of $STD_{off\text{-}on}$ spectrum is shown 10×.

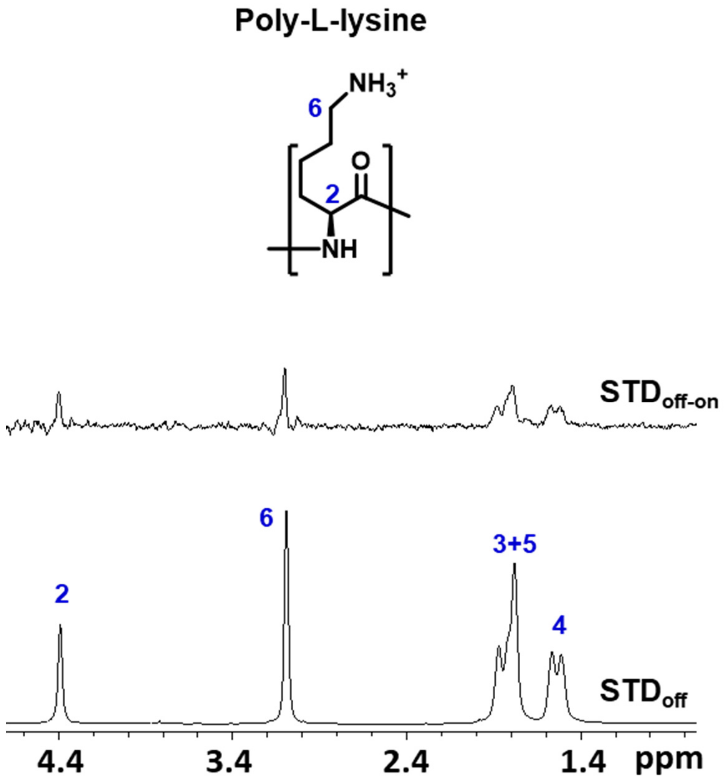

Figure S19. IF-STD spectra (750 MHz, 298 K, on-saturation at -0.22 ppm, saturation time 3 s) of poly-L-lysine (PLL 45 kDa, 10 mg/mL in D_2O). Vertical scale of $STD_{off\text{-}on}$ spectrum is shown 100×.

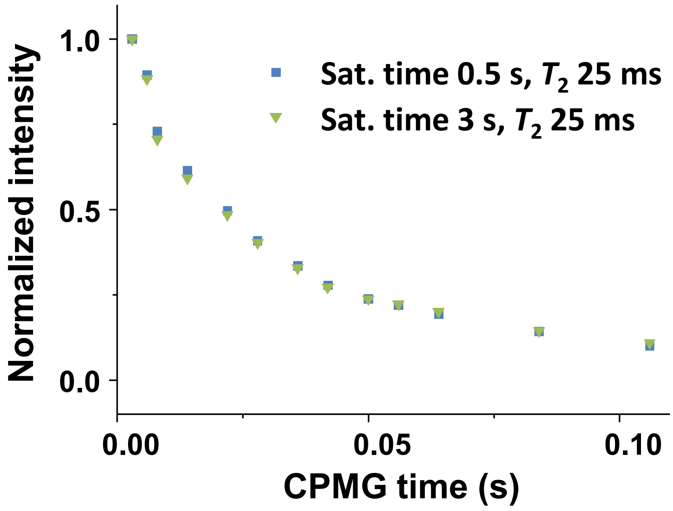

Figure S20. Normalized ^{1}H intensities (I/I0) for the H2 of CS (80 kDa, DA 14, 10 mg/mL in pD 4.5 acetate buffer) as a function of the CPMG time in hybrid IF-STD_{off}-CPMG (750 MHz, 298 K). Saturation times of 0.5 and 3 s led to ^{1}H T_2 of 25 ms.

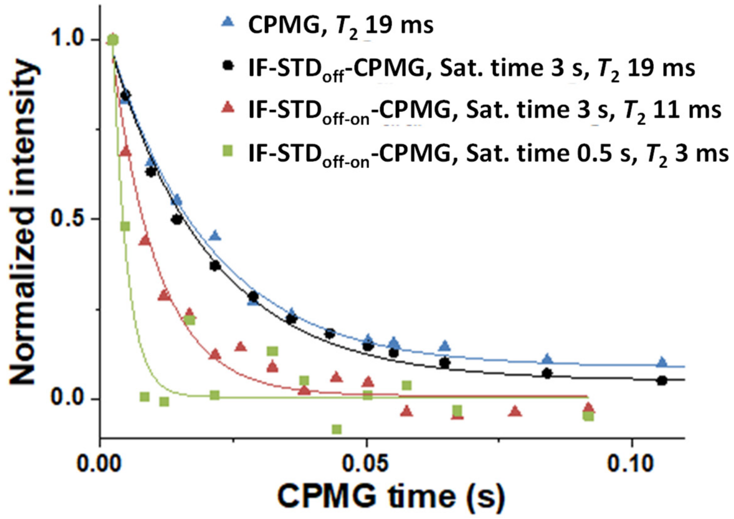

Figure S21. Normalized ^{1}H intensities (I/I0) for the H3-H6 of CS (80 kDa, DA 14, 10 g/L in pD 4.5 acetate buffer) as a function of the CPMG time in conventional CPMG and hybrid IF-STD-CPMG (750 MHz, 298 K).

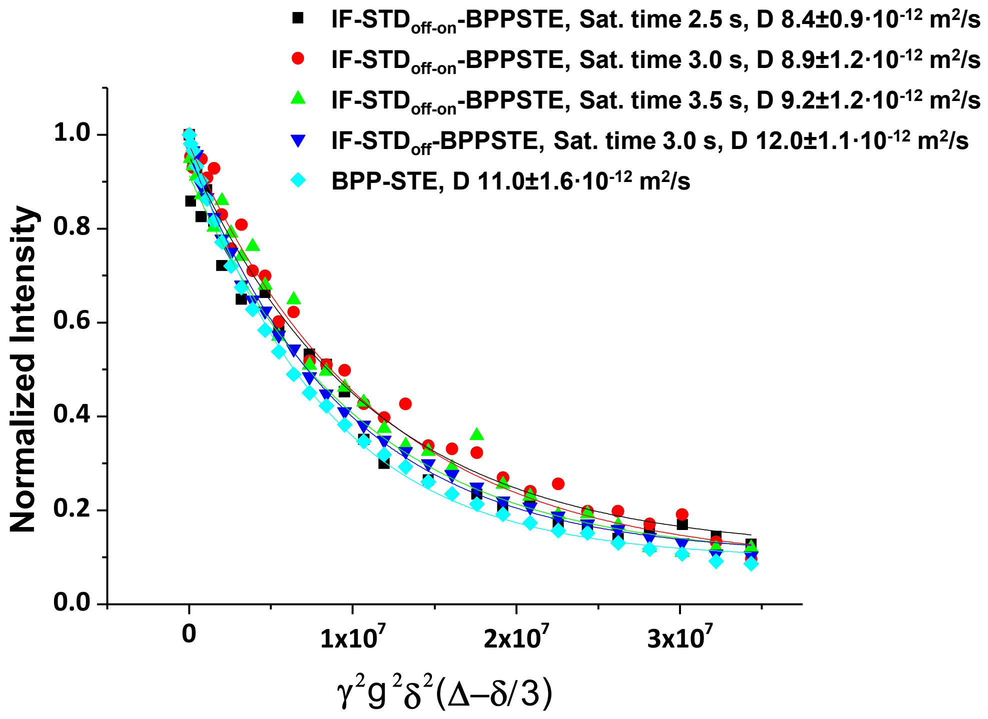

Figure S22. Stejskal-Tanner plots for the H2 of CS (80 kDa, DA 14, 10 g/L in pD 4.5 acetate buffer) obtained via conventional BPPSTE and hybrid IF-STD-BPPSTE (750 MHz, 298 K).

4. References

1. Fernandez-Megia, E.; Novoa-Carballal, R.; Quiñoá, E.; Riguera, R. Optimal routine conditions for the determination of the degree of acetylation of chitosan by 1H-NMR. *Carbohydr. Polym.* **2005**, *61*, 155-161.

2. (a) Tømmeraas, K.; Vårum, K. M.; Christensen, B. E.; Smidsrød, O. Preparation and characterisation of oligosaccharides produced by nitrous acid depolymerisation of chitosans. *Carbohydr. Res.* **2001**, *333*, 137-144. (b) Mao, S.; Shuai, X.; Unger, F.; Simon, M.; Bi, D.; Kissel, T. The depolymerization of chitosan: effects on physicochemical and biological properties. *Int. J. Pharm.* **2004**, *281*, 45-54.

3. (a) Carr, H. Y.; Purcell, E. M. Effects of Diffusion on Free Precession in Nuclear Magnetic Resonance Experiments. *Phys. Rev.* **1954**, *94*, 630-638. (b) Meiboom, S.; Gill, D. Modified Spin-Echo Method for Measuring Nuclear Relaxation Times. *Rev. Sci. Instrum.* **1958**, *29*, 688-691.

4. (a) Wu, D.; Chen, A.; Johnson Jr., C. S. An Improved Diffusion-Ordered Spectroscopy Experiment Incorporating Bipolar-Gradient Pulses. *J. Magn. Reson., Ser. A* **1995**, *115*, 260-264. (b) Johnson Jr., C. S. Diffusion ordered nuclear magnetic resonance spectroscopy: principles and applications. *Prog. Nucl. Magn. Reson. Spectrosc.* **1999**, *34*, 203-256. (c) Antalek, B. Using pulsed gradient spin echo NMR for chemical mixture analysis: How to obtain optimum results. *Concepts Magn. Reson.* **2002**, *14*, 225-258.
